# Identifying the facilitators and barriers of a healthy transition for adult children when an older parent is transferred to a nursing home: a narrative approach

**DOI:** 10.1186/s12875-026-03419-x

**Published:** 2026-06-19

**Authors:** Maylen Reiersøl Dahl, Grethe Eilertsen, Siri Tønnessen, Truls Juritzen

**Affiliations:** 1 Department of Nursing and Health Sciences, Faculty of Health and Social Sciences, University of South-Eastern Norway, Grønland 58, Drammen, 3045 Norway; 2 USN Research Group of Older Peoples’ Health, Faculty of Health and Social Sciences, University of South-Eastern Norway, Grønland 58, Drammen, 3045 Norway; 3 Department of Nursing and Health Sciences, Faculty of Health and Social Sciences, University of South-Eastern Norway, Raveien 215, Borre, 3184 Norway; 4 Head of Research, Lovisenberg Diaconal University College, Lovisenberggata 15B, Oslo, 0456 Norway

**Keywords:** Qualitative research, Narrative, Adult children, Nursing home, Healthy transition, Transfer, Municipal healthcare

## Abstract

**Background:**

As the health of an older parent deteriorates, their transfer to a nursing home becomes inevitable to ensure that they receive safe and sufficient care, with the decision to transfer often being made and implemented within a matter of days. Family caregivers such as adult children remain actively involved in providing care after their parent’s transfer to a nursing home, and are often described as an ‘invisible workforce’. However, the impact of this situation on the health and well-being of adult children is rarely acknowledged. This study aimed to characterize the transition experiences of adult children during the weeks after their older parent’s transfer to a nursing home, which is referred to as post-transition phase.

**Methods:**

This research study formed part of a larger qualitative project that had a narrative, longitudinal, prospective design. We conducted 22 narrative interviews with 8 adult children of older parents on 3 occasions in 2022. Narrative analysis was used to structure and systemize the generated data.

**Results:**

The findings are presented as two narratives of one daughter (‘Susan’) and one son (‘Phillip’) that exemplify common characteristics shared by all of the participants. They illustrate the facilitators and barriers of a healthy transition—defined as a positive and successful transition outcome—during the complex post-transition phase. Although sharing similar contextual circumstances, their narratives diverge with Phillip’s account conveying a sense of calm and relief, while Susan’s narrative reflects stress and tension. These exemplar narratives of adult children highlight how personal, organizational and social factors both facilitate and hinder a healthy transition.

**Conclusions:**

The transfer of an older parent to a nursing home is often emotionally challenging for adult children. Healthcare personnel can help reduce the burden during the transition by supporting role mastery, subjective well‑being and well-being of relationships. Promoting the well‑being of adult children may have positive implications for their older parents as well as the healthcare services and society as a whole.

**Supplementary Information:**

The online version contains supplementary material available at https://doi.org/10.1186/s12875-026-03419-x.

## Introduction

As the life spans of people increase worldwide, maintaining their good health becomes increasingly important, including so that they can continue to make significant contributions to their families and communities [1]. This situation is increasing the burden on healthcare services worldwide that are also facing challenges such as a shortage of qualified professionals due to difficulties in recruiting and retaining healthcare personnel [2–4]. This has resulted in substantial reliance on family caregivers providing a significant proportion of long-term care [2, 4–6]. This reliance is often framed as a cost-saving strategy, and the profound impact on the health and well-being of family caregivers is rarely acknowledged [2].

While the benefits of an older parent remaining in their own home in later life are widely recognized, even when they require assistance [7, 8], once their health deteriorates beyond a certain level, a transfer to a long-term facility such as a nursing home can become necessary to ensure that they receive safe and sufficient care [9, 10]. This transfer is often seen as a last resort and is typically a forced and inevitable decision initiated by a critical acute incident, safety reasons, medical needs or quality of life, and is often initiated by family caregivers rather than by the older person [10–12]. Family caregivers are often required to arrange the transfer within a matter of days, with such rapidly arranged moves resulting in both patients and their families feeling disempowered [10, 13–15]. One study showed that even after transferring an older parent to a nursing home, some family caregivers remain involved in providing care, such as for the older parent’s self-care needs, mobility needs and household activities; these caregivers have been characterized as an ‘invisible workforce’ [16].

Informal care may become an important source of support for some individuals due to the low affordability and availability of formal long-term care services [6]. The situation in Scandinavian countries such as Norway is generally distinctive due to their healthcare systems being publicly funded and highly focused on principles of equality, social justice, universalism and equal access to a minimum standard of healthcare services regardless of financial status, gender and social class [17, 18]. Norway has a population of approximately 5.6 million, including around 270,000 people aged 80 years and older. In 2024, 21,500 individuals in this age group experienced a long-term placement in a nursing home, meaning that the majority of older people live in their own homes, and around 70,000 of those aged 80 + years receive home-based care services [19, 20]. Nevertheless, informal care still plays a significant role in countries with a strong welfare state since this does not eliminate the reliance on family caregivers. Instead, their role is redefined, by supplementing formal care due to perceived gaps in staff-provided services, as well as the desire to contribute to the older adult’s well-being [9, 21–24].

While caregiving relationships among close relatives may encompass a wide range of kinship ties, the present study specifically focused on the relationship between adult children and their care-dependent older parents, which reflects the central role played by adult children in informal care in Norway [25]. When an older parent is physically transferred to a nursing home, their adult children experience a parallel transition journey [11], with healthcare personnel such as nurses often acting as the primary caregivers of the patient, and their families undergoing a transition [26]. This results in the individuals needing to display new responses, skills and strategies to cope with their new relationships and roles, which alters their views of self and the world. If the person cannot understand and adapt to the new role, they might experience insufficiency or shortcomings [27–29]. Adult children often experience a filial responsibility to provide care as their parents age, which can be perceived as a privilege or even an obligation [30]. When transitioning from the primary caregiver for home-dwelling parents to visitors at a nursing home [31], adult children need to redefine, reframe and transition into their role in the new context and situation. The resulting uncertainty and the possibility of insufficient information increase the difficulty of navigating and establishing their new roles [9]. Adult children may additionally experience feelings of sadness and guilt due to not them fulfilling their older parent’s wish to remain in the home, emotions that can persist for months or even years after the actual transfer. Adult children may also experience relief due to them feeling reassured that their family member is safe and cared for [11, 32].

Some adult children report that their transition experiences are successful, with this being more likely when they prepare sufficiently, are familiar with the healthcare system and other family members make substantial efforts [22]. Others experience disempowerment due to systemic barriers, insufficient involvement in decision-making and concerns about the quality of care [15]. Supporting patients and family caregivers as well as giving them opportunities to express their concerns and feelings about environmental changes may improve their transition experience [26]. At an existential level, the transfer to a nursing home often symbolizes life’s final stage and hence has a profound emotional dimension for the affected adult children [11]. The transfer additionally signifies the end of the older parent’s independence, further increasing the emotional burden on adult children [11, 22].

Most previous studies of transitional care have had retrospective designs [10, 33], which is potentially associated with participants being unable to accurately recall past events [34]. To address this limitation, the present study was designed to capture adult children’s narratives during the transitional phase as it unfolds; that is, their real-time experiences [10]. Accordingly, the aim was to characterize the real-time post-transition experiences of adult children. The post-transition phase is the period after the transfer of their older parent from their own home to a nursing home has been completed, and it extends for several weeks thereafter. This process involves adaptation, adjustment and acceptance related to their older parent’s new and unfamiliar living situation [33, 35].

In this article, the term ‘transfer’ describes the older parent’s physical move, while ‘transition’ refers to the adult children’s ongoing emotional, practical and social adjustments, since the admission of a family member to a nursing home also represents a life transition for their relatives [36]. Furthermore, the terms ‘narrative’ and ‘story’/‘stories’ are used interchangeably to provide linguistic variation.

## Theoretical framework

The concept of transition represents a broad, interdisciplinary field of research that spans diverse research traditions and fields across time. This includes van Gennep’s foundational ethnographic work on rites de passage in the early twentieth century, Turner’s contributions to anthropology through his elaboration of van Gennep’s liminal transitional phase during the middle of the twentieth century, and the conceptualization of transition theory within nursing research by Chick and Meleis late in the twentieth century [27, 37, 38]. The present study was guided by transition theory as outlined by Schumacher and Meleis [39], which is a process-focused theory relevant to clinicians, researchers and theorists [39, 40]. This theory builds on the work of Chick and Meleis [27], who defined a transition as a passage or movement from a place, state or condition to another, which originates from the Latin verb ‘transire’, meaning ‘to go across’. Schumacher and Meleis [39] expand the foundational work of Chick and Meleis by elaborating the following four types of transitions: (i) *developmental transitions*, such as changes in the parent–child relationship in later life, (ii) *situational transitions*, such as changes in physical location, for example when a family member transfers to a nursing home, (iii) *health–illness transitions*, such as changes in a person’s health necessitating a different level of healthcare and (iv) *organizational transitions*, such as changes in the wider social environment and intraorganizational structures driven by political, social and economic factors [26, 39].

The present study investigated the post-transition phase in order to identify facilitators and barriers of a *healthy transition*, defined by Schumacher and Meleis [39] as a positive and successful transition outcome that may occur across all four types of transitions. Individuals who experience healthy transition outcomes tend to share three essential characteristics: (i) *role mastery*, which is the ability to perform the new role competently and feel comfortable with its behavioural expectations, (ii) *subjective well-being*, which is a shift from feelings of stress and tension to a sense of contentment and (iii) *well-being of relationships*, which refers to the restoration and strengthening of family relationships [39]. Such outcomes may emerge at different points during the transition process, and so the outcomes of a healthy transition are not limited to the end of the transition period [39]. Facilitators and barriers to a healthy transition are influenced by personal, community and social factors. These include meanings attached to the event that initiated the transition process and/or the transition itself, expectations of the transition experience, and the level of knowledge and skills relevant to the transition. Additional influential factors include the resources in the overall environment available during the transition such as other family members and healthcare personnel, the level of planning before and during the transition, and emotional and physical well-being during the overall transition process [26, 39, 40].

## Methodology

### Design

This research study applied a qualitative approach in a narrative, longitudinal, prospective design that allowed the participants to be followed over several weeks as they navigated the transition over time, which is consistent with a transition involving different phases [26, 39]. The participants were interviewed at three distinct time points so that the time course of their transition experiences could be characterized. The study formed part of a larger qualitative project. In a previous study we investigated the pretransition phase [32], while the present study investigated the diversity and nuances of adult children’s real-time experiences during the post-transition phase. Such knowledge is essential for improving the understanding of adult children’s experiences across the different phases of the transition process [11, 41]. As noted by Sandelowski [42], the starting and ending points of such events are not fixed empirical entities, but rather are socially constructed and culturally influenced interpretations.

This article draws on narratives obtained in the interviews performed at all three time points to provide a rich and nuanced understanding of the post-transition phase. The adult children’s stories were constructed from the interactions that occurred between each participant and the first author during the qualitative interviews, reflecting a social constructionist understanding in which human ‘knowledge’ is developed, transmitted and maintained in social contexts [43, 44]. The narratives were constructed by reflecting on actions and experiences that either *support* or *hinder* the pursuit of goals and the realization of intentions. Our examinations of all of the interviews allowed us to identify the factors that could have shaped the different trajectories observed in the adult children’s narratives during the post‑transition phase. By considering the temporal dimension through past events, present circumstances and future expectations, stories can facilitate the ability to understand behaviours and meanings [45, 46].

### Study setting: inclusion criteria and recruitment procedure

Data on the adult children’s experiences that would help to fulfil the study aim were obtained using purposive sampling in a medium-sized Norwegian town [44]. The following inclusion criteria were applied when enrolling each participant: (a) the adult child had an older parent aged ≥ 75 years, (b) the adult child was registered as the closest relative, (c) the older parent had recently been granted long-term placement in a nursing home, (d) the older parent had received home-care services and (e) the older parent lived in their own apartment or house, or had experienced a short-term/emergency stay in a nursing home. The included parents were considered to be representative of a significant proportion of the individuals who enter nursing homes in Norway, who are the frailest persons requiring the most care within the municipal healthcare system [19, 20].

Case managers at the municipal healthcare purchasing office recruited seven participants through postal invitations and telephone contact, while the eighth participant was personally recruited by healthcare personnel at a short-term ward in a nursing home. These eight participants comprised four women and four men who ranged in age from their 40 s to their 60 s, with all but one of these children being married or cohabiting. Two participants were retired, while the other six were in paid employment. Written and verbal information about the study was provided to the participants by post and in person. The adult children’s signed informed-consent forms were sent by post to the first author or collected in person at the first interview.

### Data generation

The first author conducted 22 interviews on 3 occasions (see Supplementary File 1 and Table 2) at locations chosen by the participants. The interviews took place between February and September 2022, with each lasting 40 to 120 min, and they were digitally recorded and transcribed verbatim by the first author. The depth and richness of the data obtained in this study were ensured using individual in-depth interviews that provided the participants with ample opportunities to develop their unique and situated narratives through a co-constructive process with the interviewer. As Riessman [47] notes, “[…] the researcher does not find narratives but instead participates in their creation” (p. 21). To avoid participants telling stories they assumed the interviewer wanted to hear, which would threaten trustworthiness, the interviewer continuously encouraged the participants to freely share, in their own words, how they had experienced the transition.

The longitudinal design further increased the trustworthiness of the study, since each participant was interviewed at three distinct time points. This allowed the participants to elaborate and refine their stories as the transition process unfolded, thereby increasing the richness and depth of the data. In addition, the repeated interviews enabled the researcher to review key topics and to reflect on and verify her own interpretations from earlier interviews. This approach contributed to the significance of the study by facilitating detailed descriptions, complexity, and nuance [46]. Overall, we considered the data to be sufficient for developing a nuanced understanding of the participants’ situations and for establishing a solid foundation for in-depth narrative analyses.

### Narrative analysis

We used a combination of narrative analysis [45] and a trajectory approach for analysing longitudinal qualitative data [48] to iteratively structure and systemize the generated data. To ensure familiarity, all interview transcripts were thoroughly reviewed by the first and last authors, and collaboratively discussed among all of the authors. The data material was organized by initially arranging all of the interviews chronologically into timelines and matrices, which provided a longitudinal visual overview of past events, present circumstances and future expectations [49]. These preparatory steps were completed prior to deciding on the analytical phases that later constituted the overall qualitative study.

With the focus on the post-transition phase, this visual overview provided a solid foundation for interpreting the diverse yet comparable contextual circumstances of the participants [45, 50]. The stories differed in detail and richness, since while some of the participants readily adapted to the nursing-home environment, others faced significant challenges, which resulted the formation of two distinct narratives of relief and tension. During the analytic work, narratives of relief were characterized by a sense of calm and reassurance, with participants conveying that their parent was well cared for in a safe environment by competent healthcare professionals. This, in turn, appeared to foster relief and enable the adult children to re-engage in hobbies and general life activities that resembled their previous routines. In contrast, narratives of tension were marked by persistent worry and uncertainty, including fear and inadequate trust that the parent’s health needs were being met. Concerns related to issues such as medication management and access to outdoor activities contributed to the adult children having ongoing responsibilities and interest in what was happening at the nursing home, leaving them with little time for themselves.

In this regard, trustworthiness was addressed through analytic transparency, reflexive engagement and rich narrative descriptions, where credibility derives from interpretive depth and coherence. The data were scrutinized for counternarratives, and the analysis results were critically reviewed from the different perspectives of the authors based on various theories. The division into narratives involving relief and tension led to the following analytical question inspired by Polkinghorne: ‘why did this come about?’ [45]. Comparing such cases over a certain time span enabled the authors to collaboratively investigate how past experiences may have influenced the trajectory of the participants’ narratives [41, 49]. This approach was applicable to the present study, where the adult children were followed longitudinally. Consistent with the idiographic nature of narrative research [47, 51], we focused our analysis on two cases: Phillip and Susan (pseudonyms). We made a deliberate methodological choice to present only two cases since they represent two distinct narratives that capture patterns recurring across all of the adult children’s accounts. Experiences of relief and tension were particularly pronounced across all eight cases, and Phillip’s and Susan’s narratives were chosen since they contained rich, vivid and detailed accounts that captured two distinct perspectives while also containing common characteristics that were shared by the other six participants.

Both Phillip and Susan described experiences that encompassed optimistic and challenging aspects of their transition experiences, though to varying degrees, with Phillip primarily emphasizing a smooth and positive process, whereas Susan’s narrative illustrated greater difficulties and concerns associated with her mother’s transfer. Accordingly, new matrices were developed to trace the pathways of relief and tension in Susan’s and Phillip’s post-transition experiences using a trajectory-based approach [48].

Furthermore, the authors focused on selecting events and actions in the transcripts that would contribute to constructing narratives that answered the analytical question [45]. This approach also provided the researchers with renewed insights that allowed them to examine the material from new perspectives, focusing on the special characteristics of Phillip’s and Susan’s actions and revealing the uniqueness of their individual cases [45, 50]. Their narrative interviews were systematically examined to identify excerpts for constructing cohesive narratives. This was performed as an iterative process between individual excerpts and the narratives. The process of emplotment, where experiences are organized into a coherent and temporally structured whole, was subsequently used to consolidate the narrated events and actions into a structured description characterized by a beginning, middle and end [45, 52]. The researchers’ diverse experiences and perspectives in nursing—encompassing gerontology, psychiatry, ethics and healthcare service development—provided valuable insights into the analysis process that facilitated nuanced interpretations of the narratives while also challenging existing preconceptions.

### Ethical considerations

In accordance with data protection legislation in Norway, the study was preassessed by the Norwegian Agency for Shared Services in Education and Research (Sikt, registration number: 990316). Sikt concluded that the handling and processing of personal data were consistent with General Data Protection Regulation (GDPR) requirements. This research study did not require preapproval from the Regional Committees for Medical and Health Research Ethics (REK) since it was not considered as medical and health research aimed at gathering new knowledge about health and disease [53]. Although research projects in Norway that fall outside of REK’s mandate do not require a permit or committee approval, each researcher and research organization is still responsible for implementing appropriate ethical practices that are in accordance with the Norwegian Research Ethics Act [54]. It is also important to note that many Norwegian research institutions have not yet established independent ethics committees, which means that it is not possible for an independent ethical review to be performed when projects fall outside the scope of REK. This oversight structure is described in Froud et al. [55].

Permission to conduct the research study was granted by the municipal healthcare authorities in consultation with their legal experts. Ethical considerations and decisions were carefully reviewed and addressed throughout the study by collaborations between the authors and members of the USN Research Group of Older Peoples’ Health. All of the informed consents were obtained before the first interview. The participating adult children were provided with detailed information about the study, and advised about confidentiality and their right to withdraw consent at any time without facing any consequences. All transcripts were de-identified, and all names used in this study are pseudonyms. In adherence to the ethical principles outlined in the WMA Declaration of Helsinki [56], we were mindful of the potential challenges that the adult children might face when sharing personal and emotionally difficult life stories, and so took great care to treat the participants with respect, sensitivity and care throughout the interviews.

### Rigor and reflexivity

A pilot interview was conducted that led to minor refinements of the interview guide, including reordering the sequence of questions and adding follow-up questions to provide greater depth when necessary. The study design enabled us to follow the changing perspectives of the participants throughout the transition process, and allowed the adult children to share their stories in depth. This approach aligns with recommendations to track participants’ journeys across transitional phases in order to better understand their experiences [11]. Follow-up questions were employed between interviews to build on previous responses and identify the emerging themes more thoroughly. Field notes were logged systematically after each interview to facilitate reflections on both the experiences and observations within the interview settings. Furthermore, the analysis, narrative construction and text revision were carried out collaboratively by the authors to ensure that the research process was rigorous. As Finlay [57] emphasizes, the characteristics of researchers inevitably influence the collection, selection and interpretation of qualitative data, and this influence was addressed by the present authors throughout the study by incorporating reflexive practices and investigating assumptions and preconceptions, including collaborative discussions among the authors to ensure agreement and to minimize potential bias in the research process.

## Findings

The study findings are presented by first introducing the narratives related to how Phillip and Susan experienced the post-transition phase. This is followed by a shared emplotment reflection, with a commentary chapter presenting the thoughts and interpretations of the authors that highlight the similarities and differences among Phillip’s and Susan’s narratives [45].

Phillip is a man in his 40 s who works in an administrative position and lives with his wife and their two teenage children, while Susan is a woman in her 60 s who has retired from an administrative position, resides with her husband and has two adult children. Both have assumed primary responsibility for their ageing parents over a long time period. Their older parents were described as active, vital, social and engaged individuals. Following a long period of gradually declining health, which included some hospital stays and short-term placements in nursing homes, both parents eventually transferred to long-term placements in nursing homes due to the occurrence of acute incidents. Although Phillip and Susan share comparable contextual circumstances, their narratives have markedly different implications.

### Phillip’s story: a phase of relief after nursing-home admission

Reflecting on past years, Dad was always mentally active, solving crosswords, sudoku puzzles and playing chess. We also enjoyed forest walks together and he continued driving his car. Over time his interest faded. I noticed signs a few years ago that he was starting to ‘get old’. Not confused, but lacking initiative and drive. He stopped forest walks and he no longer sent messages or called. Eventually, I assumed primary responsibility for his household. It felt like his ageing happened overnight, almost like flipping a switch where one system after another shuts down. In the end, he could barely waddle his way to the bathroom, spending a lot of time in his armchair watching TV. There was a lot of uncertainty and worry: ‘How much longer will this old man hold on? How much can his health decline before it all comes to an end?’ Shortly thereafter, an acute incident in his home led to a hospital admission followed by a short-term placement in an institution. Currently, Dad is in a long-term placement at a nursing home, but he continues to face challenges. Even simple tasks, like sitting in his armchair, are challenging because he struggles to sit upright. When I try to assist, he resists, most likely due to difficulties with balance. However, now I know he is well taken care of because he looks tidy. I feel more at ease mentally knowing he is safe. The first long-term placement he resided at, I got the impression that he was just being ‘stored’; there was little stimulation, no healthcare personnel visible and most doors were closed. However, at the present long-term placement, the doors are mostly open, and I can see how the other patients’ rooms have been furnished like small living rooms or apartments. So, then we also made sure that Dad has a nice home there. I buy him a few things he enjoys, such as beer, snacks and cognac. He even gets his cognac served in a sippy cup, and the cognac bottle is steadily emptying [laughs]. The healthcare personnel frequently stop by to check on him, give him a pat on the shoulder, and ask how he’s doing; they are very friendly and helpful. He also participates in activities, like singing and piano sessions. This past weekend, they even hosted a family caregiver event organized by a local retirees’ association. So, there’s always something happening! Nevertheless, I would have appreciated receiving an information flyer when we arrived, outlining what they expect from me as a relative, what I need to remember, what we need to provide, what meals he is served, visiting hours, the daily routines in the unit and what I can expect from them. Just some general information that they could leave in his room for relatives to access. Now, I am wondering what the future will hold. One of the employees said that she didn’t think he would last very long. That wasn’t a surprise, because I’ve been thinking the same thing myself; how bad can it get before…before it’s the end? This has all happened so quickly, from being able to walk on his own to barely being able to sit upright by himself. I wonder how steep this downward curve can get before he hits the bottom.

### Susan’s story: a phase of tension after nursing-home admission

Looking back, it’s hard to believe that just 5 months ago she was living in her own home, was social with friends, doing her own laundry and moving around her apartment, and now she’s…completely exhausted. Mum and I also went on shopping trips together, as she loved to dress up nicely. She has always been well dressed and with styled hair, always concerned about having a proper appearance. Even though Mum has been ill over the past few years, things have happened suddenly in the past weeks, with hospitalization due to an acute incident in her home. It’s been all downhill from there. It doesn’t seem like the personnel in the nursing home have read previous reports about Mum, because some think she has been in a wheelchair for years. Now, when visiting her at the nursing home, she says odd things. I find myself wondering, ‘Is this real, or is it something she dreamt?’, which leaves me feeling uncertain. This is why improved communication and better information sharing by the healthcare personnel would be incredibly helpful. When I step out into the hallway to ask for information, no one is there. And when I finally do manage to ask, I can’t help but feel like I’m being a bother. They don’t say I am, but that’s how it comes across. I shouldn’t have to constantly dig and ask for information every single time. It would be incredibly helpful if we were provided with a standard sheet upon arrival, covering key points such as rights, responsibilities and general information about daily life in the nursing home. My daughters stay actively involved, but I wish other family members would share more of the responsibility with me. Most of the tasks tend to fall on me, as they always have, and I would have appreciated greater recognition for my efforts. So, I constantly find myself worried, feeling like I am leaving her in an unpredictable and unsafe situation. It’s like I’ve become a mother to my own Mum, an exhausting responsibility, having to follow up on her needs as she struggles to express herself. What troubles me the most is how slowly the healthcare personnel respond to the alarm clock, especially when she’s unable to move or take care of herself. Once, my daughter found Mum crying in her raised bed. She was half-dressed, lying in her own urine, while the personnel performed the shift handover. That moment was the breaking point for me, and I worry whether she’s receiving the care she truly needs. It often feels like they forget this is her home…used cloths, towels and wound-care supplies are left scattered across tables and counters. They dress her in stained clothing that carries an unpleasant, strong ammonia-like smell. I can’t help but reflect on whether they would allow such conditions in their own homes. What I find particularly disheartening is that there’s no opportunity to reconsider the assigned placement. Thinking ahead, I know that she would never manage in her home, but at the same time, I feel guilty and sad because she is where she is. She just wants to die; she can’t stand being there. She has spent most of her time in bed lately, being so tired she just lies with her eyes closed. The situation is moving towards the end, which makes it even more painful to know that she didn’t receive the care she deserved at the start of her stay. It is heartbreaking to observe that people who dedicated their lives to building up our country must spend their final days like this. Don’t get me wrong, I believe most of the healthcare personnel are doing the best they can, even though there’s still room for improvement. To be fair, things have improved since the start of her stay, especially with the healthcare personnel who caused the most issues being on holiday. When the day comes that she passes, it will undoubtedly be painful, but at the same time, it will bring her the relief she longs for. Honestly, I can’t truly relax until everything comes to an end.

### A shared emplotment reflection

Both Phillip and Susan described a gradual decline in their older parent’s health, which eventually led to an acute incident indicating the need for long-term care in a nursing home. For a long time both of them had been responsible for their older parent’s household and experienced ongoing grief as they watched their older parent becoming increasingly frail and distant. They recollect and portray a life before infirmity took over, when their parent was active, vibrant and energetic. Their narratives diverged after the physical transfer. Phillip observed that his father was thriving in the nursing home and conveyed a sense of relief and ease. Phillip now had more time and energy for everyday tasks and could focus on being a son, as he enjoyed furnishing his father’s room. The care burden that once defined the home situation was now managed by competent and attentive healthcare professionals, relieving Phillip of an expectation for him to contribute to this work, thus allowing him to focus on the relationship with his father rather than on his healthcare needs. He received strong support and recognition from both the healthcare personnel and his siblings during this period. Phillip explained that the organizational, structural and interpersonal aspects of the nursing home were supportive, even though he would have appreciated more information at the beginning of his father’s stay. In contrast, Susan’s narrative indicates that her mother was not having positive experiences in the nursing home, and that her expectations of a safe and supportive environment had not been met. She mentioned issues such as insufficient information being exchanged both between the healthcare services in general and among the nursing-home personnel in particular. She had neither the time nor the energy to focus on everyday mother–daughter activities because she felt compelled to perform caregiving tasks that the healthcare personnel were not completing. For example, Susan described having to clean up after the healthcare personnel and responding to alarms that were not attended to. This prevented her from being a daughter to her mother, instead feeling like a mother to her own mother and being responsible for taking on the role as a healthcare personnel member. This resulted in her narrative conveying a sense of persistent unease and emotional distress. The narratives of Phillip and Susan reflect back and forth on the past and present situations, and they both convey a common feeling of uncertainty about what lies ahead. Looking back, they were surprised at how rapidly their older parent’s health has declined and the realization that the vital and active parents they once knew were no longer there. Each of them reflected on their older parent’s upcoming death, and wondered how much time remains. However, one distinction between their narratives is that Phillip experienced a well-supported and well-facilitated transfer to the nursing home, which allowed him to come to terms with the situation, whereas Susan’s experienced less support. Phillip’s narrative conveys a greater sense of calm and acceptance regarding the situation and what lies ahead, whereas Susan’s account is marked by unease and restlessness. Nevertheless, Susan gradually experienced an improvement in her relationship with the nursing home and with the overall situation.

## Discussion

The results in this study indicate that the transfer of a parent to a nursing home marks an individual transition process for adult children, with this process influenced by factors that can either facilitate or hinder the occurrence of a healthy transition defined as a positive and successful transition outcome [39]. To shed light on the transition complexity and the efforts made by the adult children, this discussion addresses the three characteristics of healthy transition outcomes: (i) role mastery, which means performing the new role competently, (ii) well-being of relationships, encompassing relief and control in the nursing home and (iii) coming to terms with the inevitable, which is a dimension of subjective well-being. After addressing the main findings for these three characteristics, we move to a broader discussion that interprets these findings through the lens of transition theory.

As emphasized by Meleis et al. [26], transitions are complex and multidimensional processes that are often affected by critical incidents or turning points—these periods of heightened vulnerability demand the attention, expertise and knowledge of healthcare personnel. In the context of the present study, Robinson and Fisher [11] note that few studies have applied transition theory to investigate the transition experience of adult children when an older parent is transferred to a nursing home. The present discussion addresses this gap by considering how adult children cope during the post-transition phase, and considering what factors facilitate and hinder a healthy transition during this period for the adult children [39].

### Role mastery: performing the new role competently

It was found that nursing-home placement became inevitable as the health of Susan’s and Phillip’s older parents deteriorated. Although sharing similar experiences of change and loss related to the transfer, their stories diverged as their parents entered the nursing home: while Phillip’s account conveyed reassurance and relief, marked by a sense of control in navigating his role in the nursing home, Susan’s narrative revealed tension and stress as she struggled to establish her role within this new setting.

In addition to the situational transition, where adult children manage the abrupt transfer of a parent from their own home to a nursing home [10, 15, 39], our findings indicate that they experience a significant developmental transition as family relationships and roles change during the transfer period [32, 39]. Aligning with previous research, we found that the role of the adult children as caregivers began years before the nursing-home admission [9, 29]. However, the change process during the post-transition phase reflects an adaptation in their caregiving responsibilities, since providing care in the home differs fundamentally from caregiving in a nursing home, where they are no longer the primary caregiver [35, 36, 58]. According to transition theory, a key indicator of a healthy transition is role mastery, where individuals can perform a role competently and feel comfortable with the behaviours required from them in the new context. This may include developing confidence in the nursing home’s culture and routines, employing personal coping strategies, and recognizing the support provided by healthcare personnel [36, 39]. Research indicates that family caregivers may find the role transition particularly demanding, as they grapple with letting go of their previous caregiving role and seek ways to redefine their new identity as caregivers during the transfer period [9, 35, 36, 58, 59]—this is consistent with Susan’s narrative. In turn, this may result in family caregivers being unable to provide care in the way they once did, which can induce difficult emotions [59]. Such changes often stem from newly altered circumstances, such as declining parental health, which change the parent–child relationship. This has also been demonstrated by research into intergenerational family relationships revealing marked changes in family dynamics in later life when primary responsibilities shift from parent to child, a process frequently marked by intergenerational ambivalence [30, 32, 60–62]. Nevertheless, the narratives in the present study reveal marked variations in the experience of achieving role mastery. Drawing on the transition theory of Schumacher and Meleis [39], we found that the degree to which the adult children attain role mastery represents an essential step on the pathway towards reconciliation with the overall transition, and the achievement of a healthy transition.

Nursing-home admissions are often approached as a functional process that simply involves transferring a person from one place to another, rather than as a lived and felt experience [36]. Emotional and social support is frequently considered less important than organizational and managerial factors [36], despite transition theory and other previous research indicating that this period involves a wide range of emotions [11, 32, 39]. As Larkin and Milne [58] emphasize, emotional labour—where individuals regulate and manage their own emotions to create a desired emotional state in others—occurs in both paid and unpaid caregiving contexts [58, 63, 64]. Those authors illustrated the unpaid caregiving context using the example of family caregivers in long-term care, who may suppress their own emotions to create specific emotional states in their relative, which may damage their own well-being. For example, caregivers might hide their concerns about and difficulties encountered in the nursing home in order to avoid upsetting their family members [58]. Patients and their family caregivers are more likely to experience healthy transitions when they receive appropriate support and have opportunities to express their feelings and concerns about the environmental changes that they experience [26, 39]. While the present study found that some participants did feel supported and able to voice their concerns about the new environment, others perceived a lack of such opportunities, highlighting inconsistencies that may hinder healthy transitions.

### Well-being of relationships: encompassing relief and control in the nursing home

Susan’s and Phillip’s narratives illustrate different perceptions of the nursing home. Phillip expresses satisfaction with how the nursing home is organized, highlighting positive relationships with healthcare personnel and opportunities to maintain his father–son bond, which contrasted with his father’s first long-term placement, where he felt that his father was just being ‘stored’ while receiving little stimulation. In comparison, Susan reports disappointment and unmet expectations with the poor nursing-home organization, cooperation and coordination becoming an additional burden that meant she could no longer be just a daughter.

Schumacher and Meleis [39] report that organizational transitions involve changes in the environment, such as in the wider social environment driven by political, social and economic factors, as well as in institutional levels such as within organizational structures and dynamics. We found that organizational transitions have implications both for the older parent and for those in close relationships with them, specifically their adult children. Individual resources such as education and job stability do not necessarily make it easy to interact with a healthcare organization, with research showing that some family members face significant challenges with bureaucratic processes [12, 65]. Beyond individual resources, transition theory indicates that perceived resources in the environment can either facilitate or hinder a healthy transition [39]. Research has indicated that insufficient support, formal healthcare resources and information can significantly increase the burden that people experience during transfers between healthcare services [9, 35, 40]. In the present study, Phillip’s positive relationship with the healthcare personnel and family members represented an important supportive resource, whereas Susan encountered greater challenges. We found that the lack of an appropriate organizational structure can contribute to feelings of powerlessness, which starkly contrasted with the sense of control that Susan had experienced in her parent’s home. This suggests that these transitions should be understood both as processes involving the affected individuals and as contextual phenomena influenced by structural conditions. Preparation and planning can facilitate a healthy transition, such as knowing what to expect and having strategies, knowledge and skills to manage the transition itself [26, 40]. The present study found that a healthy transition can be hindered by a lack of organizational structures resulting from considerable variations between nursing homes, as well as a lack of interpersonal knowledge about the caregiving transition itself [26, 39]. This raises the question of whether being admitted to the most-suitable nursing home is determined simply by luck and coincidences, rather than representing a qualified choice based on knowledge of the characteristics of particular nursing homes.

According to transition theory, the meanings attributed to an acute incident that initiates the transfer process can either hinder or support a healthy transition for the adult children [26]. Similarly, at an organizational level, fostering integration and positive relationships within the broader social network and community is essential to prevent the transition producing experiences of social isolation, rumours and suspicion [39]. The societal stigma associated with the transfer to a nursing home can often delay the process until it becomes unavoidable due to an acute incident [10]. This stigma may discourage an individual from expressing their thoughts and emotions, leaving them to manage their individual transition process in silence, which is a profoundly lonely experience [26]. The emotional burden is even higher due to the transfer marking the end of the older parent’s independence, caused by their physical and cognitive decline and an inability to manage daily tasks [11, 22, 65]. This loss may also be perceived by the older individual as imposed rather than self-chosen, reinforcing their sense of lost independence [65]. Accordingly, transitions can lead to disagreements and family disruptions that in turn may negatively impact the well-being and quality of existing intergenerational family relationships [32, 39, 61], and reduce the likelihood of a healthy transition [39].

### Coming to terms with the inevitable: a dimension of subjective well-being

The present findings indicate that Susan and Phillip were gradually coming to terms with the reality that they might soon lose their parents, prompting reflections on what the future holds. The narratives indirectly reveal that the older parent’s transfer marks the onset of a difficult period. Phillip felt reassured knowing his father was in the nursing home, content with the situation by expressing relief and feeling supported. While Susan revealed a greater degree of unease and emotional turmoil, her account indicates that she was gradually coming to terms with the nursing-home placement.

Aligned with the transition theory of Schumacher and Meleis [39], the narratives of Susan and Phillip illustrate how their everyday lives were influenced by witnessing and navigating their parent’s cognitive and physical decline. Factors such as meanings attached to the event, expectations, knowledge, skills, level of planning, emotional well-being, physical well-being and resources within the overall environment may influence how adult children experience the overall transfer of their parent and their parallel transition journey [39]. While these factors might not be explicitly articulated by the participants, they can be identified implicitly from the narratives and they provide a theoretical lens for interpreting their experiences. In this regard, Phillip’s account reflects a sense of subjective well-being, which is an essential component in achieving a healthy transition that is grounded in his confidence that he acted appropriately when his father was admitted to a nursing home. Conversely, Susan’s narrative reveals persistent stress and tension that was not alleviated nor replaced by a sense of well-being [39]. For many family caregivers, the transfer to a nursing home marks the end of their parent’s independence, and realizing this often increases the associated emotional burden [11, 22]. Susan’s and Phillip’s narratives reveal a process of coming to terms with what has been lost, while adjusting to a new reality. Phillip had progressed towards reconciliation, and his narrative contains indicators of a healthy transition. Susan’s narrative contains elements of the same process, but she was not quite there yet. This suggests that some individuals will not achieve a healthy transition outcome within the expected timeframe, as defined by role mastery, subjective well-being and well-being of relationships [39], with this instead remaining as an ongoing process.

Adult children may become increasingly aware of their parent’s vulnerability during the transfer process. The literature suggests that a transfer to a nursing home can represent an existential life crisis for the older person that potentially alters their sense of identity, self- perception and life goals [66]. This may, in turn, induce existential reflections about life and death. Vandersman et al. [59] demonstrate that family caregivers often begin experiencing anticipatory grief well before the death of a family member, possible even during the initial stages of their physical and cognitive decline. Older patients in Norway died after spending a median of 1.46 years in nursing homes in 2024 [67], illustrating that nursing homes are not only temporary accommodation but often also the parent’s last home—for most, the transfer represents the final stage in life [11].

While the transition theory of Shumacher and Meleis [39] has offered valuable insight into the key dynamics of transitions, our findings suggest that adult children’s narratives lack a clear end point and stability. Similar patterns have been found in research into transitions among young people with complex support needs [68]. It is therefore possible that the adult children included in the present study were moving into a new transition stage that extended beyond the scope of our data or were beginning a new transition process. Our adult children had navigated at least three overlapping and simultaneous transitions during the post‑transition phase: (i) moving away from what was, (ii) through what is and (iii) towards what is yet to come. Rather than manifesting as separate sequential steps, these transitions appear intertwined and ongoing.

### Strengths and limitations

One methodological strength of this study was its longitudinal approach, which allowed us to identify how the participants’ situations and their utilization of the healthcare service changed over time [49]. Real-time accounts that capture experiences as they occur provide a unique perspective into the experiences of adult children. Unlike research studies with retrospective designs, those with real-time and prospective designs are not influenced by the passage of time, which may change perceptions of different situations and alter memories [10, 34]. One limitation of this study was the homogeneity of the participants, which restricts the transferability of the findings to other groups of adult children with diverse ethnic backgrounds, lower socioeconomic statuses or poor employment situations. However, it also offers a valuable lens for critical reflection, allowing us to compare the experiences of privileged groups relative to the challenges faced by disadvantaged groups, as documented in other literature.

### Recommendations for further research

What constitutes a healthy transition outcome during the post‑transition phase still need to be clearly defined, particularly among adult children with diverse backgrounds. Future research should employ sampling strategies that include such groups, since their participation will provide a broader understanding of what facilitates or hinders a healthy transition outcome across more-diverse populations. Furthermore, Meleis et al. [26] point out that it can be difficult—or even impossible or counterproductive—to set strict boundaries on the time span of certain transition experiences. We therefore acknowledge that healthcare professionals are faced with the complex task of supporting adult children, particularly given our concern about whether transition theory fully captures the complexity inherent in this life event. As described above, it appears difficult to clearly delineate a transition process or to determine precisely where one trajectory ends and a potential new one begins. This conceptual ambiguity warrants further empirical and theoretical investigations.

The present study illustrates that the post-transition phase is far from a definitive end point, instead indicating that the transition process often continues well beyond a few weeks after admission to the nursing home and the finalization of the transfer. The 8-week period of data generation in this study was therefore not sufficient to fully capture or address the complexities that adult children face. When the last interview was finished, they seemed to enter a new phase of preparation for the next transition, which is the eventual death of their parents. This underscores the need for further research with longer timeframes. Although we cannot draw definitive conclusions about its duration, this period appears to be both intense and long. We acknowledge that gender is one perspective that is not explicitly developed in this article. However, some insight into the possible effects of gender were obtained. Susan articulated her caregiving responsibility more explicitly and reported less recognition and support from healthcare professionals than did Phillip, who appeared to receive greater acknowledgement. With due analytical caution, these patterns suggest the presence of gender-related differences. This is consistent with the findings for other family caregivers, such as husbands and wives as reported by Melilla et al. [69]. Further research is needed to elucidate these perspectives.

### Implications for clinical practice

This study suggests that unique individual and contextual factors influence the likelihood of achieving a healthy transition during a complex, long and person-specific transition process. As Groenvynck et al. [10] emphasize, healthcare personnel should play a guiding role throughout the transition when an older parent is transferred to a nursing home, to ensure continuity of care during an otherwise fragmented healthcare process. Each adult child manages the transition in their own specific way, which the narratives of Phillip and Susan illustrate clearly—although they share similar contexts, they move towards a healthy transition at different paces. Their narratives show that such variations have important implications for the organization and delivery of healthcare services. It is essential that healthcare personnel understand the factors that will enable a healthy transition. Transition theory highlights factors such as role mastery, subjective well‑being and well-being of relationships as central to achieving a healthy transition. Healthcare personnel can foster this by actively providing the individuals with realistic expectations and practical strategies for managing the associated changes [26].

We propose that supporting family caregivers throughout the transition that they experience during the transfer can strengthen collaboration and improve the overall quality of care provided to the older parent. We suggest providing emotional support for enabling families to come to terms with the situation. This requires acknowledging the unique journey experienced by each adult child and understanding that the process cannot be rushed. Support should be individualized, since transition experiences during the earlier phases will result in different pathways. The participants in these processes are not generic cases or averages, but instead have distinct and profoundly diverse perspectives. Healthcare personnel can critically evaluate their clinical practices while focusing on healthy transition outcomes, and researchers can contribute to a deeper understanding of what constitutes effective and successful transitions [39].

## Conclusion

The transfer of an older parent to a nursing home is often perceived as a challenging period that is emotionally and cognitively demanding for adult children. This study has revealed that these challenges vary significantly between individuals and that adult children produce nuanced narratives influenced by reflections on their parent’s earlier active and independent life, the present nursing‑home organization, and their expectations for the future. Efforts by healthcare personnel aimed at minimizing these challenges should focus on reducing the associated burden and, importantly, preventing additional stress and tension during this already-difficult time. This study suggests that experiencing a healthy transition—characterized by role mastery, subjective well‑being and well‑being of relationships—may sustain the health and well‑being of adult children over time. In turn, this can have positive effects not only for the adult children but also their older parents, the healthcare services and society as a whole.

## Supplementary Information


Supplementary Material 1.


## Data Availability

None of the data generated and analysed during this study are publicly available due to privacy and ethical considerations for ensuring participant confidentiality. Upon reasonable request, anonymized data can be obtained from the corresponding author.
