# Supplementary material for: Identifying the facilitators and barriers of a healthy transition for adult children when an older parent is transferred to a nursing home: a narrative approach
Source: BMC Prim Care. 2026 Jun 19;27:323. doi: 10.1186/s12875-026-03419-x (PMC13531918; doi:10.1186/s12875-026-03419-x)
Supplement: Supplementary file 1 [file 12875_2026_3419_MOESM1_ESM.docx]

**Table 2*:*** *Questions that guided the narrative interviews during the* *pre-, mid- and post-transition phases*

| ^1^**First interview, after the long-term placement was granted** | **Second interview, performed when the physical transfer was anticipated to occur, approximately 4 weeks after the long-term placement was granted** | **Third interview, performed when the physical transfer has occurred, approximately 8 weeks after the long-term placement was granted** |
| --- | --- | --- |
| **(a)** What has been your experience with the situation so far? | **(a)** It is approximately 4 weeks since your mother/father was granted long-term placement in a nursing home. How are you doing now? | **(a)** It is approximately 8 weeks since your mother/father was granted long-term placement in a nursing home. How are you doing now? |
|  |  |  |
| **(b)** How are you doing now after your mother/father was recently granted long-term placement in a nursing home? | **(b)** What has been your experience from the day your mother/father was granted long-term placement up to today? | **(b)** What has been your experience with the transfer since our last interview up to today? |
|  |  |  |
| **(c)** How have you experienced collaboration with healthcare personnel to date? | **(c)** How have you experienced collaboration with healthcare personnel since our last interview? | **(c)** How have you experienced collaboration with healthcare personnel since our last interview? |
|  |  |  |

^1^ Some of the transfers occurred before a long-term placement was granted, as an emergency/short-term stay due to acute health issues that made remaining in their home unsafe
